# An Update on the Mutual Impact between SARS-CoV-2 Infection and Gut Microbiota

**DOI:** 10.3390/v14081774

**Published:** 2022-08-15

**Authors:** Shaoshuai Li, Yang Zhou, Dongmei Yan, Yanmin Wan

**Affiliations:** 1 Department of Infectious Diseases, Shanghai Key Laboratory of Infectious Diseases and Biosafety Emergency Response, National Medical Center for Infectious Diseases, Huashan Hospital, Shanghai Medical College, Fudan University, Shanghai 200040, China; 2 Shanghai Public Health Clinical Center, Department of Laboratory Medicine, Shanghai 201508, China; 3 Key Laboratory of Microecology-Immune Regulatory Network and Related Diseases, School of Basic Medicine, Jiamusi University, Jiamusi 154000, China; 4 Shanghai Public Health Clinical Center, Department of Radiology, Shanghai 201508, China

**Keywords:** SARS-CoV-2, gut microbiota, gut–lung axis, cross-reactive immunity

## Abstract

The gut microbiota is essential for good health. It has also been demonstrated that the gut microbiota can regulate immune responses against respiratory tract infections. Since the outbreak of the COVID-19 pandemic, accumulating evidence suggests that there is a link between the severity of COVID-19 and the alteration of one’s gut microbiota. The composition of gut microbiota can be profoundly affected by COVID-19 and vice versa. Here, we summarize the observations of the mutual impact between SARS-CoV-2 infection and gut microbiota composition. We discuss the consequences and mechanisms of the bi-directional interaction. Moreover, we also discuss the immune cross-reactivity between SARS-CoV-2 and commensal bacteria, which represents a previously overlooked connection between COVID-19 and commensal gut bacteria. Finally, we summarize the progress in managing COVID-19 by utilizing microbial interventions.

## 1. Introduction

The gastrointestinal (GI) tract houses a huge micro-ecosystem containing trillions of microorganisms that play essential roles in health and diseases [1,2,3]. Growing evidence shows that the gut microbiota is associated with infectious diseases and inflammation [4,5,6], e.g., multiple studies demonstrate that gut microbiota can regulate immune responses against respiratory tract infections, such as tuberculosis and influenza [7,8,9,10,11,12,13,14]. Moreover, it has also been shown that the gut microbiota is involved with chronic lung diseases via the gut–lung axis [15,16], including asthma, chronic obstructive pulmonary disease, and cystic fibrosis [17,18,19,20,21]. Since the outbreak of COVID-19, the link between gut microbiota and COVID-19 severity has been intensively studied [22,23,24,25,26]. In this review, we will focus on discussing the reciprocal impacts of gut bacteria and COVID-19, thereby characterizing the role of commensal gut bacteria in the pathogenesis of SARS-CoV-2 from a holistic point of view.

## 2. The Causal Link between COVID-19 and Gut Dysbiosis

Airborne infection is the major route of SARS-CoV-2 transmission [27,28]. However, the respiratory tract is not the only site that SARS-CoV-2 can infect. The intestine can also be an important site of infection, as enterocytes express high levels of ACE2 and TMPRSS2 [29], which have been identified as the two major cell surface molecules mediating SARS-CoV-2 infection [30]. It has been proven by several studies that the virus can replicate in the intestine [31,32,33,34]. Meanwhile, GI complications [24,35,36,37], in addition to respiratory-tract infection symptoms [35], such as fever, fatigue, and dry cough, can be frequently observed among COVID-19 patients. The most common GI symptoms accompanied by SARS-CoV-2 infection are anorexia, diarrhea, nausea, vomiting, and abdominal pain/discomfort [38,39,40,41,42], which can occur in either combination or alone. For example, an analysis of symptoms in 411 COVID-19 patients shows that 42 patients reported gastrointestinal symptoms (10.2%), including nausea (18, 4.3%), vomiting (16, 3.8%), diarrhea (15, 3.6%), or abdominal pain (5, 1.2%) [41]. Although most COVID-19-associated GI symptoms are mild, patients with GI symptoms are reported to have a significantly increased risk of severe COVID-19 compared with patients without GI symptoms [39].

Multiple potential mechanisms might be involved with COVID-19-associated GI symptoms. First, the SARS-CoV-2 infection of gut mucosa results in the endocytosis of the apical ACE2 protein [43,44], a key regulator of dietary amino acid homeostasis and innate immunity in the intestine [45,46], thereby prompting inflammatory responses in the gut. As SARS-CoV-2 infection itself can trigger inflammatory responses in the intestine [47], the dysfunction of the ACE2 may exaggerate the inflammation and lead to the damage of intestinal mucosa [48], as proven by the elevated fecal calprotectin [49] and increased infiltration of lymphocytes in the lamina propria of the GI tract [50]. Second, SARS-CoV-2 infection of the intestinal epithelium can impair the expression of the ACE2, which might cause gut dysbiosis [43,45,51]. The ACE2 forms a heterologous complex with the amino acid transporter B^0^AT1, which controls the uptake of tryptophan in the intestine [45]. This amino acid regulates the expression of antimicrobial peptides through the mTOR pathway [45]. Downregulation of the ACE2 can cause decreased intestinal absorption of tryptophan and lead to the dysregulation of antimicrobial peptides, intestinal leakage, and dysbiosis [45,52,53]. SARS-CoV-2-mediated intestinal leakage may lead to systemic elevation of bacterial lipopolysaccharide and peptidoglycan, further worsening gastrointestinal inflammation [54]. ACE2 knockout mice exhibited altered gut microbiota and developed more severe dextran sulfate sodium-induced colitis compared to wild-type control mice [45]. Third, SARS-CoV-2 infection of the respiratory tract may regulate the immune status of the digestive tract via the “gut–lung axis”, as exemplified by the observation that respiratory-influenza infection induced CCR9^+^ CD4^+^ T cells can be recruited to the small intestine and cause intestinal injury by disturbing the intestinal microbiota composition [55]. In addition, another study using an influenza-infected mouse model demonstrates that influenza-induced IFN-Is, produced in the lungs, can lead to gut dysbiosis and increase host susceptibility to secondary Salmonella transmission, inhibit intestinal immunity, and promote intestinal inflammation [56]. The mechanism underlying the impact of the respiratory-tract infection, from SARS-CoV-2, on the gut microbiota has not been clarified. However, it is suggested that inflammatory cytokines and hypoxia can drive gut dysbiosis during the viral infection [57]. In addition to the above potential mechanisms, some of the COVID-19-associated GI symptoms, such as diarrhea, are related to the use of large quantities of antibiotics [36,58] or reduced food intake [59,60].

It remains unresolved which above mechanism plays a major role in the COVID-19-associated GI symptoms. But, it is quite clear that all these mechanisms can lead to gut dysbiosis, which is actually more prevalent than GI symptoms among hospitalized COVID-19 patients [24,61]. The causal relationship between COVID-19 and gut dysbiosis has been verified by animal experiments, which have shown that SARS-CoV-2 infection alters the composition of gut microbiota in mice [62,63], hamsters [64,65], and nonhuman primates [66]. The COVID-19-associated gut dysbiosis is characterized by the enrichment of opportunistic pathogens and the decrease in beneficial symbionts [67]. Compared with healthy persons, patients with COVID-19 have significantly reduced microbial diversity, lower abundances of the anti-inflammatory bacteria (such as Lachnospiraceae, Eubacterium, and Faecalibacterium prausnitzii), and enriched abundances of opportunistic pathogens (such as Streptococcus, Rothia, Veillonella, and Actinomyces) [24,68]. In addition, patients with COVID-19 also have increased proportions of opportunistic fungal pathogens, such as Aspergillus flavus and Aspergillus niger, in feces [61]. Notably, the COVID-19-associated alteration of the gut microbiota occurs irrespective of whether the patient was medicated, and the symptoms can last long after disease resolution [25,69]. The alteration of gut microbiota caused by the SARS-CoV-2 infection is different from that of the H1N1 infection [68]. The abundance of certain common opportunistic bacteria, such as Enterococcus and Enterobacteriaceae, can serve as diagnostic biomarkers for critical COVID-19 [26].

## 3. The Reverse Impact of Gut Dysbiosis on COVID-19 Disease Progression

The aforementioned evidence shows that SARS-CoV-2 infection impacts the composition of gut microbiota; conversely, the alteration of gut microbiota composition is also found to correlate with increased severity and mortality rates among hospitalized COVID-19 patients [24,25,26,70,71,72,73,74,75]. The abundance of certain bacteria, such as Enterococcus [71] and Bacteroides [75,76], correlates positively with the severity of COVID-19. While, Faecalibacterium, which is suggested to be a marker of health, is negatively correlated with the severity of COVID-19 [24,77]. Moreover, the relatively high incidence of severe COVID-19 found in elderly patients [78,79,80,81] might also be partly explained by the decreased diversity of gut microbiota [82], because there is a possible link between the gut microbiota diversity and the clinical outcome of COVID-19 [23]. This notion is corroborated by a multivariate analysis showing that the Shannon diversity index of gut microbiota is significantly associated with COVID-19 severity [83]. Although the association between gut dysbiosis and the severity of COVID-19 has been observed in different clinical settings (Table 1), the causal impact of the gut microbiota on the severity of COVID-19 has not been fully clarified. A preprint study provides the first evidence that the gut dysbiosis caused by the SARS-CoV-2 infection can lead to a gut-to-blood translocation of microorganisms, suggesting a direct role for gut dysbiosis in enabling secondary bloodstream infections during COVID-19 [71]. Meanwhile, Edwinson et al. suggested that commensal microbiota might play a key role in regulating intestinal ACE2 expression using a humanized mouse model [84].

In addition to microbial translocation, gut microbiota can alleviate or aggravate viral infections via other mechanisms [88,89]. On the one hand, microbial products at the entry site can bind with viruses to enhance their stability and infectivity; on the other hand, the microbiota can inhibit viral entry via regulating local pH and host immune responses [90]. Given that gut microbiota or their products are less likely to interact directly with SARS-CoV-2 in the respiratory tract, their impact on the infection in the lung is more likely driven by the indirect modulation of host immunity. The alteration of gut microbial composition can influence inflammatory responses outside the gastrointestinal tract through diverse mechanisms [91]. First, it has been found that the perturbation of gut microbiota can stimulate the immune system to release cytokines such as IL-1β, IL-2, IL-10, TNF-α, and IFN-γ, which may exacerbate the severity of COVID-19 [92,93,94]. As it has been suggested, mortality associated with COVID-19 is mainly caused by enhanced cytokine and chemokine production. This contributes to virally induced hyper-inflammation, referred to as the “cytokine storm” [95,96], where the overproduction of proinflammatory cytokines may exacerbate the severity of COVID-19 [97]. Mechanistically, the reduced abundance of probiotics, such as butyrate-producing bacteria [26,87,98], may undermine the anti-inflammatory effects mediated by regulatory T cells [99]. At the same time, the enrichment of Escherichia and Shigella could lead to systemic inflammation [98]. Additionally, the downregulation of the ACE2 by the SARS-CoV-2 infection may lead to the increased activation of the renin-angiotensin system (RAS), which may cause systemic vasoconstriction and systemic inflammatory response syndrome (SIRS) [100]. Conversely, blocking the renin-angiotensin pathway has been shown to be able to alleviate the SARS-CoV-2 spike protein-induced acute lung failure in mice [101].

Second, gut microbes can affect physiological and pathological immune responses in the airways through neural, endocrine, immune, humoral, and metabolic pathways [102,103,104], which are collectively described as the gut–lung axis [105]. The gut–lung axis is bidirectional, but most of the current evidence suggests that gut microbiota could most likely regulate lung homeostasis, which is exemplified in patients with chronic gastrointestinal diseases, such as irritable bowel syndrome (IBS) and inflammatory bowel disease (IBD), who have a higher prevalence of pulmonary diseases [106,107,108]. Via the gut–lung axis, the gut microbiota can impact the production of type I interferons (IFNs) in the lung [12,13,14], which are well known to control viral infections, including SARS-CoV-2 [109,110,111]. Microbial metabolites such as deaminated tyrosine (DAT, derived from flavonoid and amino acid metabolism) and short-chain fatty acids (SCFAs, the end products of dietary fiber fermentation by commensal bacteria) have been shown to be critical in regulating the anti-virial immunities in the respiratory tract [12,13]. SCFAs exert anti-inflammatory, anti-antitumor, and antibacterial effects by inhibiting histone deacetylase (HDAC) and activating the G protein-coupled receptor (GPCR) [112,113]. They can also strongly reduce the release of several proinflammatory chemokines through regulatory T cells, including CCL3, CCL4, CCL5, CXCL9, CXCL10, and CXCL11 [114]. In addition, butyrate and propionate can inhibit the expression of lipopolysaccharide (LPS)-induced cytokines such as IL-6 and IL-12p40, displaying a strong anti-inflammatory effect [114,115]. The reduced abundance of SCFA-producing bacteria observed in the gut microbiota of COVID-19 patients may be one of the key mechanisms leading to severe clinical outcomes, according to previous studies [26,87,98,116]. Moreover, the impaired capacity to synthesize short-chain fatty acids and L-isoleucine by the gut microbiome of COVID-19 patients continues even after the remission of the disease [94]. Additionally, it has also been found that the upper gastrointestinal microbiota affects the development of the airway microbiota [117], which plays an immediate role in calibrating the alveolar immunity of COVID-19 patients [118]. Taken together, the facts listed in this section imply that the gut microbiota plays an active role in determining the clinical progression of COVID-19.

## 4. Direct Interaction and Immune Cross-Reactivity between SARS-CoV-2 and Commensal Bacteria

Available data have shown that the commensal bacteria and their products can interact directly with a variety of viruses to either promote or suppress viral infections [119,120,121,122]. Lipopolysaccharides (LPSs) and peptidoglycans (PGs) are the major microbial products that have been frequently observed to interact directly with viruses [119,123,124,125,126,127]. For example, the binding of an LPS to a poliovirus promotes virion stability and cell attachment [128]. The mouse mammary tumor virus (MMTV) can integrate a TLR-4 into its envelope to bind the bacterial LPS. The LPS bound to the MMTV stimulates the secretion of IL-10 through TLR-4 signaling, and IL-10 allows viral persistence through negative immune regulation [126,127]. The interaction between the SARS-CoV-2 S protein and the LPS has also been observed and proven to be able to boost cytokine responses in human peripheral blood mononuclear cells [129]. More intriguingly, a recent study demonstrates that the SARS-CoV-2 spike S1 subunit can inhibit the biofilm formation by Streptococcus pneumoniae and Staphylococcus aureus [130], suggesting that coronavirus infections may promote these opportunistic pathogens to resume a more virulent planktonic lifestyle. Moreover, although it has not been experimentally validated, an amino acid blast analysis suggests that proteobacteria may secrete homologues of the TMPRSS2 and the ACE2 peptidase domain [131], which may presumably inhibit SARS-CoV-2 infection via blocking the binding of the spike protein to the receptors.

Based on amino acid sequence analyses, another study indicates that common human pathogens and vaccines, such as Meningococcal B and combination vaccines for diphtheria, tetanus, and pertussis (DTP vaccine), may potentially induce cross-reactive immunity to SARS-CoV-2 [132]. This hypothesis is partly supported by a clinical observation that pre-existing antibodies acquired from childhood vaccinations or past infections of Rubella, Pneumococcus, and Bordetella pertussis may confer some protection against COVID-19 [133]. It has also been proven that commensal gut bacteria may facilitate the induction of neutralizing antibodies [134,135,136] and cross-reactive T cell responses [137,138] against viruses such as HIV-1. In a recent study, we provide the first experimental evidence that pre-existing antibodies targeting a conserved linear epitope on S2 (1147-SFKEELDKYFKNHT-1160) cross-react with commensal gut microbial antigens [139]. Specific monoclonal antibodies against the epitope are proven to cross-react with diverse antigens of gut bacteria, such as the HSP60 and HSP70 proteins derived from *E. coli* [139]. Our finding is corroborated by a subsequent study suggesting that this conserved spike epitope shares sequence homology to proteins in commensal gut microbiota and can prime immune responses in humans [140]. Of note, this is not a phenomenon only observed with respect to S2-specific antibodies; accumulating evidence suggests that RBD-specific antibodies [141] and T cell responses, cross-reactive to SARS-CoV-2 [142,143,144], can be primed by commensal gut bacteria. The pre-existing cross-reactive immunities elicited by commensal bacteria may shape the host’s immune responses after infection or vaccination; however, their exact role in controlling SARS-CoV-2 transmission and infection needs to be further specified.

## 5. The Microbiota Mediated Interventions for COVID-19

Microbiota-based interventions (such as diets, probiotics, Chinese herbs, and fecal microbiota transplantation) have been used in the clinical treatment of various human diseases (such as diabetes, ulcerative colitis, Crohn’s disease, and certain viral infections) [145,146]. Considering the significant impact of gut microbiota on the course of COVID-19, modulating the composition of gut microbiota is considered a possible method for treating SARS-CoV-2 [147,148].

Several approaches are exploited to achieve this goal. The first approach involves regulating the composition of the gut microbiota via dietary interventions. Diets have been shown to play an important role in shaping gut microbiota [146,149,150]. For example, glycated pea proteins increase the intestinal commensal bacteria (Bifidobacterium and Lactobacillus) [149], and the high-fiber diet can alter the ratio of Firmicutes to Bacteroidetes, which can exert anti-inflammatory effects by increasing short-chain fatty acids (SCFAs) [150]. The high-fiber diet has been shown to be able to improve gastrointestinal symptoms of COVID-19 by increasing the SCFAs-producing bacteria (such as Oscillibacter, Sellimonas, Bifidobacterium, Blautia, Lactobacillus, Faecalitalea, Anaerofustis, and Eubacterium) in the gut [151]. In addition, vitamin D supplements are shown to improve clinical symptoms by reducing inflammatory cytokine levels [152], which is partly because vitamin D can modulate the composition of gut microbes [153,154].

The second method involves regulating the gut microbiota via the supplementation of probiotics. The SARS-CoV-2 infection leads to a decrease in commensal bacteria such as Lactobacillus and Bifidobacterium, which can affect innate and adaptive immune responses to prevent and mitigate bacterial and viral infections [155,156,157]. Observations in mice infected with influenza A virus (H1N1) show that treatments with probiotic strains (Enterococcus faecalis and Bifidobacterium) can downregulate inflammatory cytokines by balancing the Th1/Th2 immune response and reducing the mortality of mice [158,159]. Clinical studies suggest that probiotics can be used to reduce inflammation by changing the composition of gut microbiota in COVID-19 patients [160], which includes the enrichment of intestinal commensal bacteria and the inhibition of opportunistic pathogens [161,162]. Moreover, it has also been found that drug therapy combined with probiotics can reduce gastrointestinal symptoms and mortalities in COVID-19 patients [163,164].

Third, regulating gut microbiota using traditional Chinese medicine (TCM) is a potential approach. It has been demonstrated that some Chinese herbs can regulate gut microbiota [165,166]. For example, extracts of Ginseng radix et rhizome rubra and Coicis semen promote the growth of probiotics (Lactobacillus and Bifidobacterium) and inhibit the growth of pathogenic bacteria (Escherichia, Staphylococcus, and Salmonella) [167]. Gegen Qinlian decocted can elevate the relative abundance of SCFA-producing bacteria, including Akkermansia, Bacteroides, Clostridium, Ruminococcus, and Phascolarctobacterium [168]. The treatment of COVID-19 with TCM via regulating gut microbiota has been proposed [166], but clinical and experimental evidence is needed to verify this notion.

Fourth, regulating gut microbiota via fecal microbiota transplantation (FMT) can be used to treat a variety of diseases related to gut dysbiosis [169,170]. It has been proven that FMT treatment can improve gut dysbiosis in recovered COVID-19 patients, especially in those with severe gastrointestinal symptoms [171].

## 6. Conclusions

**Figure 1:**
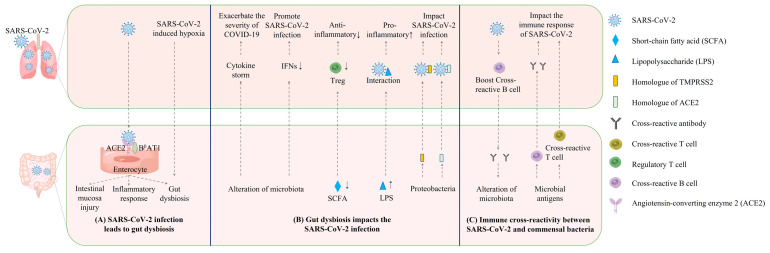
Potential mechanisms underlying the mutual impacts between intestinal microbiota and SARS-CoV-2. (**A**) SARS-CoV-2 can cause gut dysbiosis by infecting enterocytes or causing systematic hypoxia. (**B**) Gut dysbiosis can impact SARS-CoV-2 infection in the lungs via regulating immune responses in the lung or secreting homologues of ACE2 and TMPRSS2. (**C**) The microbial antigens may elicit cross-reactive antibody and T cell responses against SARS-CoV-2. SARS-CoV-2 infection may reinforce cross-reactive antibody responses against microbial antigens and thereby lead to the alteration of gut microbiota.

The SARS-CoV-2 infection can cause gut dysbiosis and GI symptoms; conversely, gut microbiota can also impact the SARS-CoV-2 infection in the respiratory tract (Figure 1). Multiple mechanisms are involved in this mutual interaction. The gut–lung axis is usually believed to be the major bi-directional connection between the airway viral infection and the gut microbiota. In addition, a few recent studies characterized the cross-reactive antibody and T cell responses between SARS-CoV-2 and gut microbiota, demonstrating that there was an alternative bi-directional link between airway SARS-CoV-2 infections and the gut microbiota. Deeper insights into this phenomenon can expand the understanding of the entanglement between airway viral infections and the gut microbiota, thereby promoting the development of new treatments for COVID-19 and other severe respiratory viral infections.

## Figures and Tables

**Table 1:** Intestinal microbial alterations and their effect in COVID-19.

(Refs.)	Intestinal Microbial Alterations	Effect in COVID-19
[24]	Faecalibacterium prausnitzii ↓	Anti-inflammatory. Inverse correlation between abundance and disease severity.
	Alistipes onderdonkii ↓	Involving in the serotonin precursor tryptophan metabolism and maintaining gut immune homeostasis. Negative correlation with COVID-19 severity.
	Bacteroides dorei, Bacteroides thetaiotaomicron, Bacteroides massiliensis, Bacteroides ovatus ↓	Downregulating the expression of angiotensin-converting enzyme 2 (ACE2). Correlated inversely with SARS-CoV-2 load in fecal samples.
	Coprobacillus, Clostridium ramosum, Clostridium hathewayi ↑	Correlating positively with COVID-19 severity. Coprobacillus bacterium upregulates the expression of ACE2.
[68]	Streptococcus, Rothia, Veillonella, Actinomyces ↑	Opportunistic pathogens. Significantly increased relative abundances in COVID-19 patients compared with those in healthy controls.
	Fusicatenibacter, Anaerostipes, Agathobacter, unclassified Lachnospiraceae, Eubacterium hallii ↓	Butyrate-producing bacteria. The abundances are dramatically reduced in COVID-19 patients compared with those in healthy controls.
[61]	Candida albicans, Candida auris, Aspergillus flavus, Aspergillus niger ↑	Significantly higher relative abundances in hospitalized COVID-19 patients compared with those in healthy controls.
[25]	Faecalibacterium prausnitzii, Eubacterium rectale, Bifidobacterium adolescentis ↓	Anti-inflammatory. These bacteria are depleted in COVID-19 patients.
	Bacteroides dorei, Akkermansia muciniphila ↑	Correlating positively with IL-1β, IL-6, and CXCL8. Enriched in COVID-19 patients.
[26]	Faecalibacterium prausnitzii, Clostridium butyricum, Clostridium leptum, Eubacterium rectale ↓	Butyrate-producing bacteria. The abundances decreased significantly in COVID-19 patients.
	Lactobacillus, Bifidobacterium ↓	Producing lactic acid, regulating immunity, and maintaining intestinal barrier function. Correlating negatively with COVID-19 severity.
	Enterococcus (Ec), Enterobacteriaceae (E) ↑	Opportunistic pathogens. Correlating positively with COVID-19 severity and the Ec/E ratio can predict death in critically ill patients.
[71]	Faecalibacterium ↓	An immunosupportive Clostridiales genus. Correlating negatively with bloodstream infection (BSI).
[72]	Bilophila, Citrobacter ↓	Correlating negatively with COVID-19 severity.
	Genus: Streptococcus, Clostridium, Lactobacillus, Bifidobacterium, ↑	The abundances increased significantly in COVID-19 patients compared with those in healthy controls.
[76]	Genus: Escherichia/Shigella, Citrobacter, Collinsella, Bifidobacterium ↑	Correlating positively with COVID-19.
	Genus: Bacteroides, Butyricimonas, Odoribacter ↓	Short-chain fatty acid (SCFA)-producing bacteria. Markedly reduced in patients with COVID-19 compared to healthy controls.
[75]	Butyricicoccus pullicaecorum, Clostridium ruminatium, Lachnospira pectinoschiza, Pseudobutyrivibrio xylanivorans, ↓	Completely absent in the guts of COVID-19-infected patients.
	Roseburia faecis, Lachnospira pectinoschiza, Faecalibacterium prausnitzii ↓	Short-chain fatty acid-producing bacteria. Correlating negatively with COVID-19 severity.
	Clostridium hathewayi, arabacteroides distasonis, Ruminococcus gnavus ↑	Correlating positively with COVID-19 severity.
[85]	Bacteroidaceae, Lachnospiraceae, Ruminococcaceae ↓	Producing short-chain fatty acids (SCFAs). The abundances decreased significantly in COVID-19 patients compared to those in healthy controls.
	Enterococcus ↑	Far overrepresented in COVID-19 patients developing bloodstream infections (BSIs) and admitted to the intensive care unit.
	Enterococcaceae, Coriobacteriaceae, Lactobacillaceae, Veillonellaceae, Porphyromonadaceae Staphylococcaceae ↑	The abundance increased significantly in COVID-19 patients compared to those in healthy controls.
[69]	Ruminococcus gnavus, Bacteroides vulgatus ↑	The abundances increased significantly in patients with post-acute COVID-19 syndrome (PACS) than in non-COVID-19 controls.
	Bifidobacterium pseudocatenulatum, Faecalibacterium prausnitzii ↓	Butyrate-producing bacteria. Correlating negatively with the development of PACS.
[86]	Genus: Roseburia, Megasphaer Species: Roseburia inulinivorans, Bacteroides faecis, Bifidobacterium bifidum, Parabacteroides goldsteinii, Lachnospiraceae bacterium 9143BFAA, Megasphaera sp. ↓	Correlating negatively with COVID-19 severity.
	Genus: Paraprevotella, Lachnospiraceae, Erysipelotrichaceae Species: Paraprevotella sp., Streptococcus thermophilus, Clostridium ramosum, Bifidobacterium animalis ↑	Correlating positively with COVID-19 severity.
[87]	Genus: Collinsella ↓	Inhibiting the binding of SARS-CoV-2 to ACE2, suppressing proinflammatory cytokine secretion, antioxidant, and anti-apoptotic. Correlating negatively with the mortality rates of COVID-19.
	Genus: Dorea, Fusicatenibacter ↓	Short-chain fatty acid (SCFA)-producing bacteria. Correlating negatively with the mortality rates of COVID-19

Notes: ↑, significantly increased; ↓, significantly decreased.

## Data Availability

Data sharing not applicable.
